# Cerebral cortex and hippocampus neural interaction during vagus nerve stimulation under *in vivo* large-scale imaging

**DOI:** 10.3389/fnins.2023.1131063

**Published:** 2023-03-02

**Authors:** Hanyun Xu, Tingting Jin, Rujin Zhang, Hao Xie, Chaowei Zhuang, Yanyang Zhang, Dongsheng Kong, Guihua Xiao, Xinguang Yu

**Affiliations:** ^1^Chinese PLA Medical School, Beijing, China; ^2^Department of Neurosurgery, The First Medical Center of Chinese PLA General Hospital, Beijing, China; ^3^Pulmonary and Critical Care Department, Wuhu Hospital of East China Normal University, Wuhu, Anhui, China; ^4^Department of Anesthesiology, The First Medical Center of Chinese PLA General Hospital, Beijing, China; ^5^Department of Automation, Tsinghua University, Beijing, China; ^6^BNRist, Tsinghua University, Beijing, China

**Keywords:** vagus nerve stimulation, *in vivo* imaging, hippocampus, cerebral cortex, neuronal activity, fluorescence microscope

## Abstract

**Objective:**

The purpose of this study was to study mechanisms of VNS modulation from a single neuron perspective utilizing a practical observation platform with single neuron resolution and widefield, real-time imaging coupled with an animal model simultaneously exposing the cerebral cortex and the hippocampus.

**Methods:**

We utilized the observation platform characterized of widefield of view, real-time imaging, and high spatiotemporal resolution to obtain the neuronal activities in the cerebral cortex and the hippocampus during VNS in awake states and under anesthesia.

**Results:**

Some neurons in the hippocampus were tightly related to VNS modulation, and varied types of neurons showed distinct responses to VNS modulation.

**Conclusion:**

We utilized such an observation platform coupled with a novel animal model to obtain more information on neuron activities in the cerebral cortex and the hippocampus, providing an effective method to further study the mechanisms of therapeutic effects modulated by VNS.

## 1. Introduction

Vagus nerve stimulation (VNS) is widely used as a treatment for various diseases, such as epilepsy, migraine, depression, etc. Although VNS therapy was approved by the US Food and Drug Administration in 1997 as an adjunctive therapy for reducing seizures in patients with refractory epilepsy, the therapeutic mechanisms remain poorly understood (Johnson and Wilson, 2018). The cerebral cortex and the hippocampus have been implicated as pivotal in VNS therapy (Attenello et al., 2016).

Currently, most studies on mechanisms of VNS modulation mainly employ electrophysiological techniques, fluorescence microscope coupled with neurotransmitter probe, and medical imaging equipment such as MRI, PET, and SPECT (Conway et al., 2006; Vonck et al., 2008; Bartolomei et al., 2016; Mithani et al., 2019; Collins et al., 2021). Electrophysiological techniques are characterized by achieving micro-information (e.g., electrical signaling of neurons). The advantages of medical imaging equipment lie in understanding the functional connectivity among brain regions and the blood flow and metabolism of brain regions. Fluorescence microscope coupled with neurotransmitter probe can analyze neurotransmitters released by neurons. Based on the current studies utilizing these observation technologies, the potential mechanisms of therapeutic effects induced by VNS may be as follows: (1) Anti-inflammatory effects of VNS: Inflammation is a protective response of the body to external stimuli. Nevertheless, excessive inflammation can induce or exacerbate various brain diseases such as epilepsy, depression, etc. (Mehta et al., 2018; Paudel et al., 2018). Many studies have found that VNS could produce therapeutic effects by reducing local and systemic inflammatory response, which has proved to be correlated with the modulation of peripheral release of cytokines from immune cells, blood–brain barrier (BBB) permeability, and status of microglia (Kaya et al., 2013; Kaczmarczyk et al., 2017; Chaudhry et al., 2019). (2) Effects of VNS on central nervous system: (1) Neurotransmitter of norepinephrine (NE) and Gamma-Amino Butyric Acid (GABA)-Some studies have demonstrated that VNS could promote the release of NE in locus coeruleus (LC) and basolateral amygdala (BLA), and notably enhance extracellular NE levels in the prefrontal cortex (PFC) and hippocampus, and VNS-induced antiepileptic effects appeared to be related to the concentrations of NE in the hippocampus (Raedt et al., 2011; Manta et al., 2013). Another animal study showed that VNS took effect through increasing the concentration of NE in limbic, thalamic and cortical brain regions (Landau et al., 2015). Additionally, VNS regulated cortical excitability in brain regions associated with epilepsy, and its therapeutic effects were related to the normalization of cortical GABAA receptor density (Marrosu et al., 2003). In a word, the therapeutic effects of VNS were related to the concentration of NE and GABA in the cerebral cortex and subcortical structures. (2) BDNF-TrkB pathway and neuroplasticity: BDNF, a modulator of hippocampal plasticity and neurogenesis, could play an active role in the prevention of neuronal death (Hofer and Barde, 1988). Long-term VNS induced an increase of BDNF expression in the hippocampus, which was closely related to memory enhancement (Biggio et al., 2009). Similarly, VNS could activate the BDNF signaling pathway through a7nAChR, thus enhancing axonal plasticity and improving long-term neurological rehabilitation (Li et al., 2020). (3) Electrophysiological activity: abnormal hypersynchronous discharge of the cerebral cortex was regarded as a characterized feature of epileptic seizures (Wang et al., 2021). VNS remarkably suppressed epileptiform activity in EEG recordings by enhancing the firing rate of NTS neurons. Epileptiform activities in EEG recordings were obviously inhibited by VNS enhancing the firing rate of nucleus tractus solitarius (NTS) neurons (He et al., 2013). (4) Functional connectivity of brain regions: VNS enhanced the connectivity of thalamus to the anterior cingulate cortex (ACC) and left island and increased the regional homogeneity of the right superior or middle temporal gyrus, therefore resulting in improvement of the clinical manifestations in patients with epilepsy (Ibrahim et al., 2017). (5) Cerebral blood flow: the neuroprotective effects of VNS could be correlated with the modulation of cerebral blood flow (CBF) in brain regions (Conway et al., 2006).

To sum up, the study on mechanisms of VNS modulation from a single neuron perspective is rare, possibly due to the lack of such an effective observation platform with single neuron resolution and widefield of view, real-time imaging, as well as an animal model simultaneously exposing the cerebral cortex and the hippocampus. Here, we established this observation platform as well as a novel mouse model to obtain neuronal activities in the cerebral cortex and hippocampus during VNS and provide a new viewpoint to further explore the mechanisms of therapeutic effects induced by VNS.

## 2. Materials and methods

### 2.1. Mouse

All experimental procedures were approved by the Institutional Animal Care and Use Committee at Tsinghua University, Beijing, China. We employed Transgenic mouse Ai148(TIT2L-GC6f-ICL-tTA2)-D × Rasgrf2-2A-dCre (JAX 022864, JAX 030328) expressing Gcamp6f calcium signaling in the specific layer 2/3 of the cerebral cortex for the imaging experiments. All experimental mice were purchased from Animal House of Tsinghua University. All animals were housed in a laboratory environment on a regular 12/12 h light/dark cycle at 20–22^°^C. Mice had access to *ad libitum* food and water and were individually housed after virus injection, craniotomy, and VNS cuff implantation. All experimental manipulations were conducted during the light phase.

### 2.2. Chemicals and apparatus

Isoflurane was purchased from the RWD life science company (Shenzhen, China). AAV2/9-hSyn-Flex-GCaMP6f-WPRE-pA (qTiter 1.12e9GC/ml) and AAV2/9-hSyn-Cre-WPRE-pA (qTiter 1.12e9GC/ml) were purchased from Shanghai Taitool Bioscience Company of China. A programmable stimulus isolator was used to stimulate the vagus nerve through connecting electrode cuff. A homemade optical widefield mesoscope was used for Ca^2+^ imaging with a central wavelength at 473 nm.

### 2.3. Hippocampus virus injection

For hippocampal imaging, the mouse was secured to a stereotaxic frame (the RWD life science company of Shenzhen, China) after anesthesia induction, and the whole injection procedures were performed in an aseptic environment with the mouse under 1–2% isoflurane anesthesia (oxygen flow rate: 1 L/min). An approximately 0.8 cm midline incision was made centered about 0.1 cm behind the bregma. After horizontal calibration adjustment, we placed the micro syringe needle at the bregma point and then reset the coordinates to zero. A 0.5 mm diameter hole was drilled on the right skull region above the hippocampus (coordination, AP: -2.2 mm; ML: 1.5 mm) to reach the dura, then we mixed AAV2/9-hSyn-Flex-GCaMP6f-WPRE-pA at 1:10,000 dilution (qTiter 1.12e9GC/ml, Shanghai Taitool Bioscience company, China) and AAV2/9-hSyn-Cre- WPRE-PA (qTiter 1.9e13 GC/ml, Shanghai Taitool Bioscience company, China) in a ratio of 1:1. A volume of 200 nl was injected into the right hippocampal CA1 (coordination, AP: -2.2 mm; ML: 1.5 mm DV: 1.3 mm) using a thin glass pipette and infusion pump at the rate of 50 nl/min. After injection, wait for 10 min for the virus to fully absorb, then we retracted the pipette and sutured the incision by absorbable suture (8–0). The mouse was returned to their cage for at least 2 weeks before surgical procedures.

### 2.4. Surgery procedures

The Surgical instruments were sterilized by autoclave for 30 min prior to each surgical procedure. All surgical procedures were performed in an aseptic environment with mice under 1–2% isoflurane anesthesia (oxygen flow rate: 1 L/min). Isoflurane anesthetic gas (1% in oxygen) was used for the entire duration of all surgeries. The temperature of the mouse was maintained between 36.5 and 37.5°C using a homeothermic blanket system. Craniotomy and cylinder implantation above hippocampus CA1. For widefield imaging, we cut the skin between and around the bilateral ears and eyes to expose enough skull space. After horizontal calibration adjustment, we placed the syringe (1 ml volume) needle at the bregma point and then reset the coordinates to zero. After Removing the syringe to a safe location, a trapezoid shape window (8 mm × 10 mm) was made overlying the dorsal cortex using a handheld dental drill. The skull was removed to expose the dura, and the gel foam particles soaked in sterile saline were gently applied on the dura to stop the slight bleeding. Wait until all bleeding was entirely stopped, then Carefully remove the gel foam particles not to disturb the clotting process. Two skull nails were implanted, respectively, in the front and back of the cranial window on the skull in order to secure the glass coverslip. Locating the coordinate of the hippocampus (AP: -2.2 mm; ML: 1.5 mm), the cortex above the hippocampus was aspirated until reaching the CA1 layer of the hippocampus featured of regular stripes was exposed. After the bleeding was completely stopped and the wound was cleaned, a cylinder lens was implanted above CA1 (AP: -2.2 mm; ML: 1.5 mm; DV: –1.3 mm), and then a trapezoid shape glass coverslip was laid over the cranial window and sealed with surgical glue. After 10 min, when the surgical glue around the cranial window was solid, a thin layer of dental acrylic cement was placed around the edges of the glass to solidify it. A semicircular aluminum alloy head post was placed on the edge of the cranial window, ensuring that it was parallel to the glass coverslip. It was then fixed with dental acrylic cement. We applied dental acrylic cement throughout the exposed skull surface and across a small rim of the coverslip to secure it. Cervical vagus nerve cuff electrode implantation. Two platinum-iridium wires were fixed 1 mm apart to biocompatible micro silicone tube. The end of the two lead wires was connected to pins, which were used to connect the cuff to the stimulator. After isoflurane induction, the mouse was placed in a supine position; the surgical site was shaved and cleaned with scrubs of iodophor and alcohol. Under anesthesia, a 1.5–2 cm incision was made from manubrium to jawline along the scalp midline on the ventral aspect of the neck using micro-scissors. With micro scissors and blunt-tipped forceps, the submaxillary gland and connective tissue overlying the left cervical vagus nerve was retracted, and the nerve was separated from the vessels within the carotid sheath. After using a small pair of surgical retractors to hold the muscles apart, a 4–5 mm segment of the left cervical vagus nerve was dissected from the carotid sheath. The cuff electrode was placed around the vagus nerve and secured with suture ensuring that the electrode wires had circumferential or near-circumferential contact with the nerve. The muscles were placed back in their original position, and an absorbable suture (8–0) was used to secure the cuff in position. A subcutaneous tunnel was made in between the ear and eyes from the neck incision to the top of the head, which allowed passing the cuff leads to the skull. Lead pins were fixed to the previously implanted head post using acrylic dental cement. The submaxillary gland was placed back to its original position, and finally the neck incision and the incision of the head’s subcutaneous tunnel were sutured closed.

### 2.5. Vagus nerve stimulation parameters

The animal’s head was fixed to the mouse holder. In all experiments, the intertrain interval was set at 50 s, and each pulse train lasted for 10 s. VNS was delivered by the parameter combination of 0.3 mA, 0.1 ms, and 5 Hz. The parameters occurred three times in a session of cerebral cortical and hippocampus axon imaging.

### 2.6. Data analysis

Widefield images of the cerebral cortex and the hippocampus were aligned to the Allen institute common coordinate framework (CCF) map using structural landmarks and MATLAB code developed by Drs. Matt Kaufman and Shreya Saxena. Widefield fluorescence signals were normalized pixel by pixel by the following equation ΔF/F_0_ = (F_*i*_-F_0_)/F_0_, where Fi is the raw fluorescence of the ith video frame, and F_0_ is the mean of the fluorescence baseline. Origin 8.0 was used to analyze the statistical data.

## 3. Results

### 3.1. Establishment of experimental observation platform and mouse model

#### 3.1.1. Experimental observation platform

**FIGURE 1:**
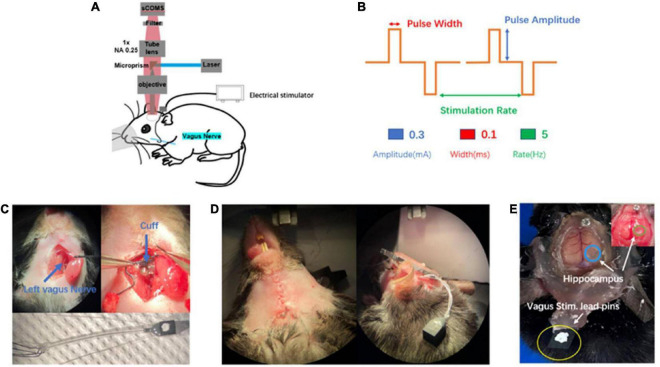
Introduction of *in vivo* wild-field imaging and vagus nerve stimulation (VNS). **(A)** Schematic diagram of *in vivo* imaging and vagus nerve stimulation, illustrating that the mouse is under overhead objective for *in vivo* calcium brain imaging and the cuff electrode is positioned around vagus nerve(blue). **(B)** Schematic of VNS pulse waveforms and parameters. Biphasic pulse was delivered in trains lasting 10 s (top). VNS was delivered by the parameter combination of 0.3 mA, 0.1 ms, and 5 Hz (bottom). **(C)** Photograph of vagus nerve cuff position (top) and bipolar VNS cuff design (bottom). **(D)** Photograph of subcutaneous tunnel made in between the ear and eyes from neck incision to the top of the head. **(E)** Photograph of the awake mouse with the fixed head post, showing simultaneous exposure of cerebral cortex and hippocampus (blue and green) as well as vagus stimulation lead pins (yellow) fixed to the rear of the head post using acrylic dental cement.

In order to further *in vivo* explore complex neuronal activities of the brain by observation of macroscopic multiple brain regions combined with microscopic neurons, we set up this observation platform (widefield mesoscope) to research the effects of VNS modulation on brain neuronal activities in awake states and under anesthesia. The observation platform’s excitation source is a CW laser (MBL-III-473–100 mW, CNI) at a central wavelength of 473 nm. The laser beam is expanded to 12 mm by a beam expander (BE) and a pair of 4f-system lenses. After being focused, then being reflected by a micro prism, the beam passes through an excitation objective and excites neuron fluorescence of the cerebral cortex and hippocampal CA1 region of the mouse. The fluorescence is collected by an epifluorescence setup including the same objective, a tube lens (MVPLAPO 1X, Olympus), a filter and an sCMOS. The FOV of the equipment is 6.6 mm and each pixel in the sCMOS corresponds to 3.25 μm on the image plane with two times magnification and 6.5 μm pixel size (Figure 1A). The equipment is characterized of single-cell resolution, widefield of view, and real-time observation.

#### 3.1.2. Establishment of mouse model for simultaneous *in vivo* imaging of cerebral cortex and hippocampal CA1 region

The experimental mouse model in previous studies was mainly related to small bone window craniotomy, exposing the cerebral cortex with a size of about 3–4 mm in diameter. There was short of an experimental animal model that simultaneously exposed large-scale cerebral cortex and hippocampal CA1 region. In our experiment, we first selected the rasgrf-Cre-Ai148D gene mouse expressing Gcamp6f calcium signaling in the specific layer 2/3 of the cerebral cortex to inject GCamp6f virus into the right hippocampal CA1 region. After 2 weeks, we performed a large-scale craniotomy and cylinder implantation above the hippocampal CA1 region, simultaneously exposing the cerebral cortex (range: 6 mm × 8 mm) and the right hippocampal CA1 region (range: 1.8 mm × 1.8 mm) (Figure 1E), which provided observation of the cerebral cortex and the hippocampal CA1 simultaneously. In a word, we established a new and original animal model for further exploring brain activities.

#### 3.1.3. Vagus nerve electrode implantation

In our experiment, we selected the left vagus nerve of the mouse for vagus nerve stimulation electrode implantation (Figure 1C top). We designed and customized the vagus nerve electrode (Figure 1C bottom) according to the size of the mouse vagus nerve. Bipolar VNS cuff can be firmly fixed to cervical vagus nerve and a subcutaneous tunnel was made in between the ear and eyes from the neck incision to the top of the head, which allowed passing the cuff leads to the rear of head post. Lead pins were fixed to the previously implanted head post using acrylic dental cement (Figure 1D). Our mouse model had the following advantages: (1) VNS cuff was firmly fixed to vagus nerve and not easy to fall off, and the electrode wire was buried under the skin, which protected the electrode wire from damage. (2) It avoided the adverse effects caused by exposed electrode wire on the daily behavior of the mouse. (3) Our mouse model could survive for a long time, providing an ideal model for various experimental designs and long-term research on brain activity. In this study, we chose a VNS stimulus parameter within the commonly used range (Figure 1B).

### 3.2. Characteristics of calcium signaling of neurons in cerebral cortex and hippocampal CA1 region in awake states

**FIGURE 2:**
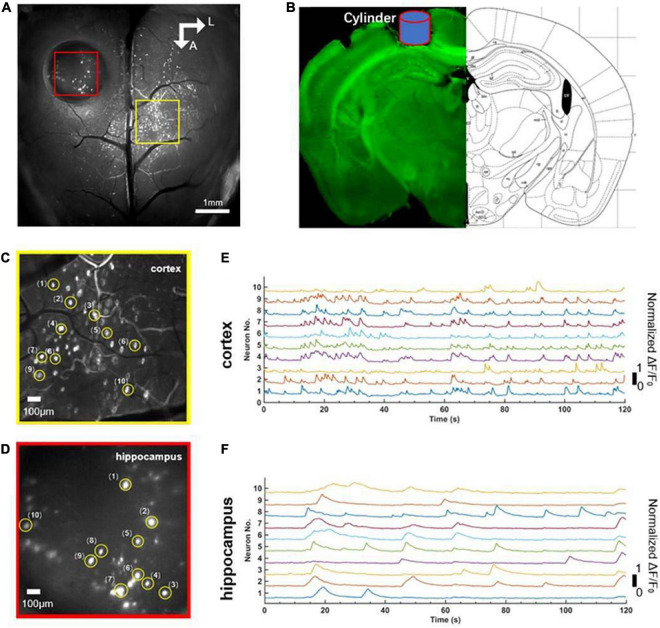
Comparison of simultaneous wide-field calcium imaging of cerebral cortex and hippocampus CA1 *in vivo* in awake states. **(A)** Photograph illustrating simultaneous wide-field calcium imaging *in vivo* of the cerebral cortex and the hippocampus CA1. Scale bar, 1 mm. **(B)** Coronal slice of the mouse illustrating the hippocampus CA1 right beneath cylinder (blue) corresponding to the red square in panel **(A)**. **(C)** A field of the enlarged view of neurons (labeled 1–10) in layer 2/3 of cerebral cortex indicated with the yellow square in **(A)**, Scale bar, 100 μm. **(D)** A field of the enlarged view of neurons (labeled 1–10) of hippocampus CA1 indicated with the red square in **(A)**, Scale bar, 100 μm. **(E)** Normalized fluorescence signals fluctuation (ΔF/F_0_) for neurons (labeled 1–10) shown in panel **(C)**. **(F)** Normalized fluorescence signals fluctuation (ΔF/F_0_) for neurons (labeled 1–10) shown in panel **(E)**.

Before the experiment, we first positioned the mouse beneath the widefield camera to make it familiar with the surrounding environment. After 10 min, when the mouse was accustomed to the surrounding environment, we used the observation equipment to obtain the characteristics of calcium signaling of neurons located in layer 2/3 of the cerebral cortex and the hippocampus CA1 of the mouse in awake states. We found that neurons of the cerebral cortex and the hippocampus CA1 had spontaneous neuronal activities (Figure 2A). Then, we randomly extracted 10 neurons from near the junction of motor cortex and somatosensory cortex (yellow square, Figure 2C) and the hippocampal CA1 (red square, Figure 2D), respectively. Further analysis showed the single neuron’s characteristic curve of Gcamp6f calcium signaling. The calcium signaling curve of neurons of the cerebral cortex and the hippocampal CA1 showed that, compared to neurons of the hippocampal CA1 (Figure 2F), neurons of the cerebral cortex had higher calcium signaling intensity and faster response frequency. The result indicated that the neurons of the cerebral cortex were more active in awake states (Figure 2E). The perfused brain slice showed that the cylinder was right above the hippocampal CA1, and the observation position was appropriate (Figure 2B).

### 3.3. Characteristics of calcium signaling of neurons in cerebral cortex and hippocampal CA1 in awake states during VNS

**FIGURE 3:**
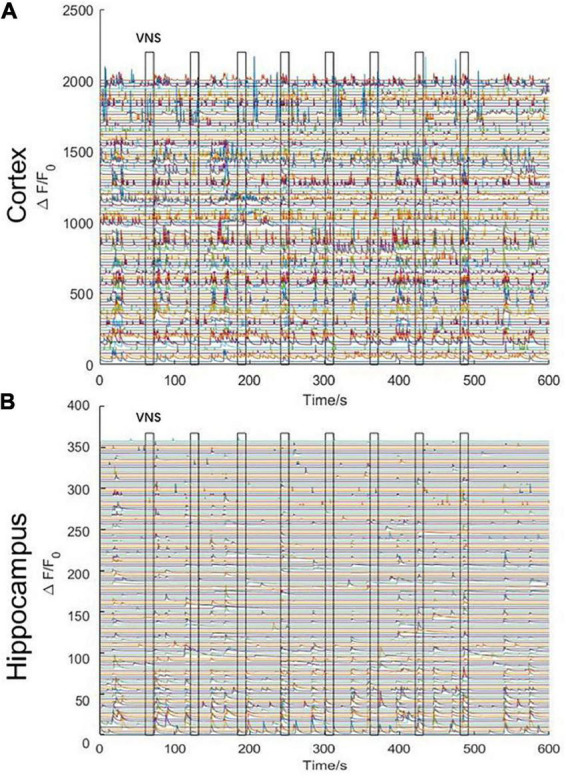
Curve diagram of calcium signaling of neurons in the cerebral cortex and hippocampal CA1 in awake states during VNS. **(A)** Curve diagram of calcium signaling of neurons in the cerebral cortex during VNS. **(B)** Curve diagram of calcium signaling of neurons in the hippocampal CA1 during VNS. Narrow gray rectangles indicate time of each VNS application (lasting 10 s). Y-axis indicates neuron numbers.

We stimulated with trains of biphasic pulses lasting 10 s. Experimental results demonstrated that neuronal activities were not tightly correlated with VNS. In other words, neuronal calcium signaling in the cerebral cortex and the hippocampal CA1 was not significantly changed during VNS, compared to the pre-VNS baseline (Figures 3A, B). Owing to being head-fixed beneath the widefield camera, the limbs and mouth of the mouse had vigorous activities, which could induce neuronal activities. Therefore, we speculated that no significant difference in neuronal activities between during VNS and pre-VNS baseline was associated with relatively small stimulation parameters or intense neuronal activities induced by body movement. That is, the intensity of neuronal activities induced by VNS was significantly lower than the intensity of neuronal activities induced by body movement. Consequently, the neuronal activities induced by VNS were covered by the neuronal activities elicited by body movement, resulting in the finding showing no significant changes in neuronal activities induced by VNS.

### 3.4. Characteristics of calcium signaling of neurons in cerebral cortex and hippocampal CA1 under isoflurane anesthesia during VNS

**FIGURE 4:**
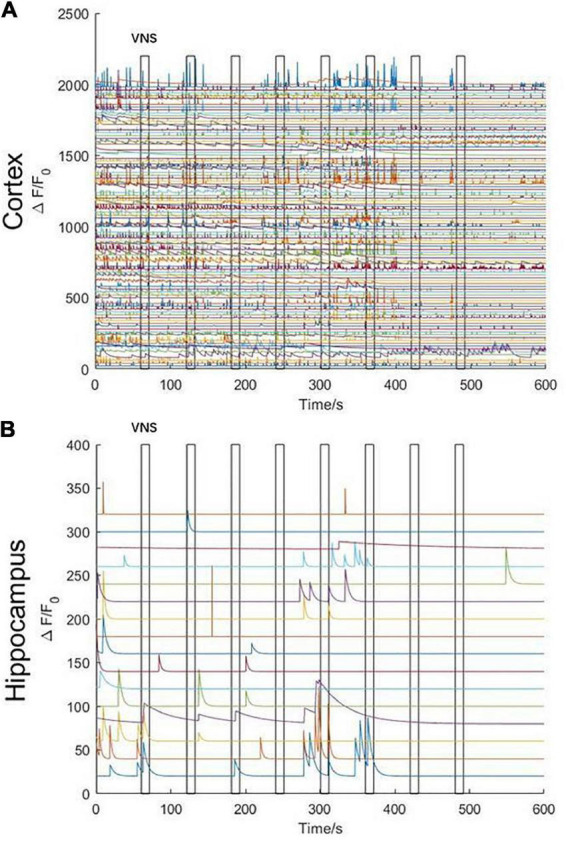
Characteristics of calcium signaling of neurons in the cerebral cortex and hippocampal CA1 under isoflurane anesthesia during VNS. **(A)** Curve diagram of calcium signaling of neurons in the cerebral cortex during VNS. **(B)** Curve diagram of calcium signaling of neurons in the hippocampal CA1 during VNS. Narrow gray rectangles indicate time of each VNS application (lasting 10 s). Y-axis indicates neuron numbers.

In order to remove the disturbance of neuronal activities elicited by noticeable limb movement in awake states, we used isoflurane anesthetics to regulate the mouse into anesthesia. The mouse inhaled oxygen through a mask connected to an anesthesia machine at a flow rate of 1.2 L/min. VNS was delivered when the mouse was under anesthesia without limb movement. In the initial stage of anesthesia, we observed that, neuronal activities in the cerebral cortex were rapidly inhibited (Figure 4A); however, neuronal activities in the hippocampal CA1 were not significantly inhibited. Meanwhile, we also found that the activities of several neurons in the hippocampal CA1 were tightly related to VNS. With the anesthesia deepening, neuronal activities in the hippocampal CA1 vanished and were no longer activated by VNS (Figure 4B). Therefore, we concluded that, at least in the hippocampal CA1 region, some neuronal activities were regulated by VNS.

### 3.5. Correlation between neuronal activities in the hippocampal CA1 and VNS

**FIGURE 5:**
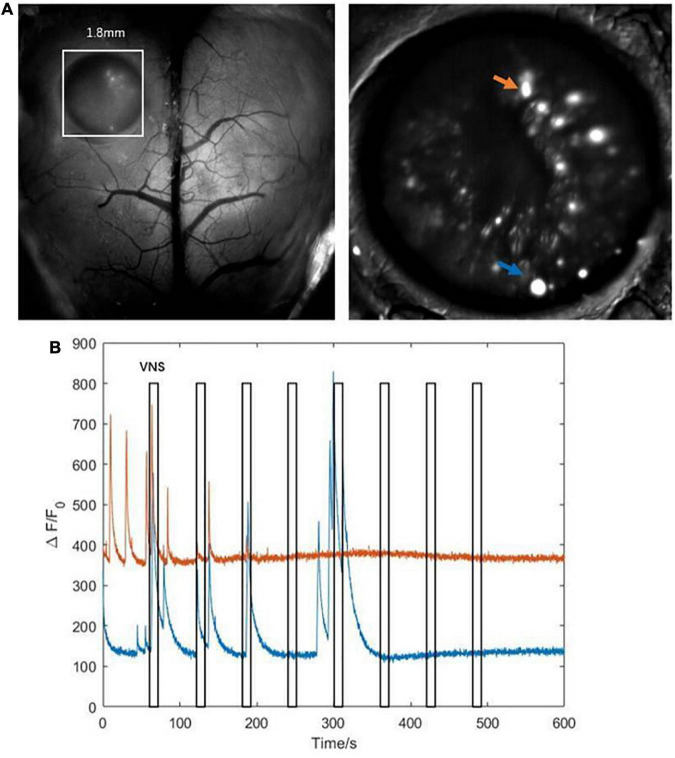
Characteristics of calcium signaling of neurons in the hippocampal CA1. (**A** left) The hippocampal CA1 region (white square: 1.8mm × 1.8mm); (right) two typical neurons extracted from hippocampal CA1 (orange arrow and blue arrow). **(B)** The curve diagram of neuronal activities of the two neurons (blue curve and orange curve corresponding to blue arrow and orange arrow shown in **(A)**, respectively). Narrow gray rectangles indicate time of each VNS application (lasting 10 s). Y-axis indicates neuron numbers.

The results above showed that the activities of several neurons in the hippocampal CA1 were tightly correlated with VNS (Figure 5A). We further extracted two typical neurons to achieve the curve of neuronal calcium signaling. We analyzed that one neuron (orange curve) showed inhibition characteristics, and the other neuron (blue curve) showed activation characteristics in response to VNS application (Figure 5B). Therefore, we speculated that different types of neurons had varied responses to VNS application.

## 4. Discussion

vagus nerve stimulation (VNS) is widely used as a treatment for epilepsy, migraine, depression, etc., since VNS therapy was approved by the US Food and Drug Administration in 1997 as an adjunctive therapy for reducing seizures in patients with refractory epilepsy (Johnson and Wilson, 2018). Despite its broad and growing application, the mechanisms by which VNS exerts its clinical benefits are still little known, especially the mechanisms in regulation of neurons. Here, we set up this observation platform, which is characterized of single-cell resolution, widefield of view, and real-time observation, to research the effects of VNS modulation on brain neuronal activities from a combination of macroscope and microscope perspective in awake and anesthetized states. Moreover, we established a new and original mouse model, exposing large-scale cerebral cortex (range: 6 mm × 8 mm) and hippocampal CA1 region (range: 1.8 mm × 1.8 cm), to simultaneously achieve *in vivo*, real-time imaging of cerebral cortex and subcortical region (hippocampal CA1). Utilizing our observation platform and mouse model, we observed that the cerebral cortex and the hippocampal CA1 existed spontaneous neuronal activities, respectively, which showed varied characteristics from each other in awake states. Moreover, we observed that neuronal calcium signaling in the cerebral cortex and the hippocampal CA1 was not significantly changed during VNS in awake states, possibly due to low-intensity stimulations or disturbance of body movement. In order to eliminate the disturbance of body movement, we achieved neuronal activities in the cerebral cortex and the hippocampal CA1 during VNS in anesthetized states. The analysis indicated that at least the activities of several neurons in the hippocampal CA1 were tightly correlated with VNS, and two of these neurons showed distinct characteristics.

### 4.1. Experimental observation platform and mouse model

Previous studies on the mechanisms underlying VNS modulation centered on either macroscopic multiple brain regions or microstructures. The macroscopic observation methods mainly utilized devices, including MRI, PET, and SPECT (Conway et al., 2006; Malbert et al., 2019; Borgmann et al., 2021), whose major advantages lay in the power to observe the functional connectivity of multiple brain regions. The microscopic observation methods primarily utilized electrophysiology and optical fiber photometry, which took advantage of observing microstructures such as cells or neurons (Jaseja, 2010; Laricchiuta et al., 2021). However, few studies effectively combined the two observation technology to decipher complex brain neuronal activities *in vivo*. Therefore, we applied this observation platform that combined macro- and microscopic observation, which was characterized of widefield of view (6.6 mm), real-time observation, and single-cell resolution, to further study neuronal activities elicited by VNS. In addition, previous studies have shown that epilepsy and depression were related to the cerebral cortex and subcortical regions (e.g., hippicampus) (Rosso et al., 2020; Wang et al., 2021). Owing to the anatomical location of hippocampus beneath the cerebral cortex, it was challenging to directly observe the hippocampus CA1 for more neuronal activities. Few studies have demonstrated a mouse model simultaneously exposing large-scale cerebral cortex and hippocampus CA1 to study brain activities. However, some studies utilized optical fiber implanted into the corresponding regions to observe synchronous neuronal activities in the motor region of the cerebral cortex and the hippocampus CA1 (Dong et al., 2020). It was challenging to obtain a larger region of neuronal signaling due to the observation scope being confined to a focal point. Our study established an experimental mouse model to simultaneously expose large-scale cerebral cortex (6 mm × 8 mm) and hippocampal CA1 region (1.8 mm × 1.8 mm). Taken together, we set up an experimental observation platform combined with a novel animal model to further explore neuronal activities in the cerebral cortex and hippocampus.

### 4.2. Characteristics of calcium signaling of neurons in cerebral cortex and hippocampal CA1 in awake states during VNS

Some studies demonstrated that cortical activation was to be dose-dependent. Low-intensity stimulation parameters that evoked little to no arousal change similarly did not elicit detectable changes in cortical excitation, whereas high-intensity stimulation parameters that elicited pupil dilation, whisking, and/or wheel movement also induced large increases in cortical neuronal calcium signaling (Collins et al., 2021; Mridha et al., 2021). In our experiment, neuronal activities in the cerebral cortex and the hippocampal CA1 were not significantly changed during VNS compared to pre-VNS baseline. Therefore, we speculated that neuronal activities in response to VNS application were weak due to relatively small stimulation parameters and the intensity of neuronal activities induced by VNS was much lower than the intensity of neuronal activities elicited by vigorous limb movement. So, there was no significant difference in neuronal activities between during VNS and pre-VNS baseline. Furthermore, the effects of VNS modulation varied among different parameters, and enhancement of performance of behavior tasks and cortical map plasticity was strongest in response to intermediate intensity of stimulation (Ghacibeh et al., 2006; Lai and David, 2021). Therefore, we concluded that there might be an individual difference in the effects of VNS modulation, and the optimal parameters were crucial to VNS application.

### 4.3. Characteristics of calcium signaling of neurons in cerebral cortex and hippocampal CA1 under anesthesia during VNS

To completely remove any contribution of limb movement-related activities to neuronal calcium signaling, we anesthetized mice with isoflurane. At the onset of anesthesia, neuronal activities in the cerebral cortex were rapidly inhibited; however, neuronal activities in the hippocampal CA1 were not significantly inhibited. Meanwhile, we also found that the activities of several neurons in the hippocampal CA1 were tightly related to VNS. With the anesthesia deepening, neuronal activities in the hippocampal CA1 vanished and were no longer activated by VNS. Therefore, we concluded that, at least in the hippocampal CA1, some neuronal activities were modulated by VNS. In previous studies, Arousal state or sleep disturbances and learning impairment related to hippocampus abnormality occurred across many forms of epilepsy, migraine, and depression, conditions for which VNS has been found to be a useful treatment option (Sedigh-Sarvestani et al., 2014; Gumusyayla et al., 2016; Sun et al., 2017). In an animal study, the finding was that the significant deviations from normal calcium dynamics in CA1 arose before (33 min, on average) the onset of motor convulsions and the intense calcium waves could directly lead to acute cellular damage in live animals (Berdyyeva et al., 2016). Combined with these previous findings, we draw a conclusion that the therapeutic effects of VNS could be partially explained by the modulation of neuronal activities in the hippocampal CA1. Additionally, we further extracted two typical neurons to obtain the curve of neuronal calcium signaling, by which we analyzed that one neuron showed inhibition characteristics and the other neuron showed activation characteristics in response to VNS application. Anatomical and physiological studies have previously demonstrated that VNS may excite cortical neurons through neuromodulatory pathways, including those releasing acetylcholine (Ach) or NE (Dorr and Debonnel, 2006; Roosevelt et al., 2006). Fibers carrying information from the vagus nerve synapse in the nucleus of the solitary tract projected to the noradrenergic LC. Then projections from LC were sent throughout the brain, including to subcortical structures, such as the basal forebrain (BF), thalamus, and cerebral cortex (Kim et al., 2016; Rho et al., 2018). Therefore, we speculated that various neurons existed in the hippocampal CA1, which had varied responses to VNS application.

## 5. Conclusion

Taken together, we utilized this experimental observation platform coupled with a novel animal model to simultaneously obtain further information from neurons in the cerebral cortex and the hippocampal CA1, which provided an effective means for further studying mechanisms of VNS modulation. Meanwhile, our experiment shed light on mechanisms of VNS modulation from a single neuron level, providing a new perspective on mechanisms of VNS modulation. Combined with previous studies’ findings, we summarized that selection of stimulation parameters was vital to the therapeutic effects of VNS modulation, and different types of neurons in the hippocampal CA1 had varied responses to VNS modulation. Next, future studies may focus on individualized VNS parameters and various types of neurons in the cerebral and the hippocampal, ultimately contributing to improved therapeutic effects of VNS modulation for clinical diseases.

## Data availability statement

The raw data supporting the conclusions of this article will be made available by the authors, without undue reservation.

## Ethics statement

This animal study was reviewed and approved by the Institutional Animal Care and Use Committee at Tsinghua University, Beijing, China.

## Author contributions

HYX, GX, and XY: conceptualization. HX, CZ, and GX: methodology. DK and RZ: validation. YZ and HYX: investigation. HYX, RZ, and GX: data curation. HYX: writing—original draft preparation. HYX, TJ, and GX: writing—review and editing. GX, HX, and XY: supervision and project administration. YZ: funding acquisition. All authors read and agreed to the published version of the manuscript.
